# Tag-Free SARS-CoV-2 Receptor Binding Domain (RBD), but Not C-Terminal Tagged SARS-CoV-2 RBD, Induces a Rapid and Potent Neutralizing Antibody Response

**DOI:** 10.3390/vaccines10111839

**Published:** 2022-10-30

**Authors:** Ting-Wei Lin, Ping-Han Huang, Bo-Hung Liao, Tai-Ling Chao, Ya-Min Tsai, Shih-Chung Chang, Sui-Yuan Chang, Hui-Wen Chen

**Affiliations:** 1 Department of Veterinary Medicine, National Taiwan University, Taipei 10617, Taiwan; 2 Department of Clinical Laboratory Sciences and Medical Biotechnology, National Taiwan University, Taipei 10617, Taiwan; 3 Department of Biochemical Science and Technology, National Taiwan University, Taipei 10617, Taiwan

**Keywords:** COVID-19, vaccine, tag-free RBD, His-tag RBD, neutralizing antibodies, Delta variant

## Abstract

Recombinant proteins are essential in the development of subunit vaccines. In the design of many recombinant proteins, polyhistidine residues are added to the N- or C-termini of target sequences to facilitate purification. However, whether the addition of tag residues influences the immunogenicity of proteins remains unknown. In this study, the tag-free SARS-CoV-2 RBD and His-tag SARS-CoV-2 RBD proteins were investigated to determine whether there were any differences in their receptor binding affinity and immunogenicity. The results showed that the tag-free RBD protein had a higher affinity for binding with hACE2 receptors than His-tag RBD proteins (EC_50_: 1.78 µM vs. 7.51 µM). On day 21 after primary immunization with the proteins, the serum ELISA titers of immunized mice were measured and found to be 1:1418 for those immunized with tag-free RBD and only 1:2.4 for His-tag RBD. Two weeks after the booster dose, tag-free-RBD-immunized mice demonstrated a significantly higher neutralizing titer of 1:369 compared with 1:7.9 for His-tag-RBD-immunized mice. Furthermore, neutralizing antibodies induced by tag-free RBD persisted for up to 5 months and demonstrated greater cross-neutralization of the SARS-CoV-2 Delta variant. Evidence from Western blotting showed that the serum of His-tag-RBD-immunized mice recognized irrelevant His-tag proteins. Collectively, we conclude that the addition of a polyhistidine tag on a recombinant protein, when used as a COVID-19 vaccine antigen, may significantly impair protein immunogenicity against SARS-CoV-2. Antibody responses induced were clearly more rapid and robust for the tag-free SARS-CoV-2 RBD than the His-tag SARS-CoV-2 RBD. These findings provide important information for the design of antigens used in the development of COVID-19 subunit vaccines.

## 1. Introduction

In the past year and at present, severe acute respiratory syndrome coronavirus 2 (SARS-CoV-2) infection has affected billions of lives around the world. A variety of vaccines have been developed for the control of coronavirus disease 2019 (COVID-19) [1,2]. According to the USA CDC, multiple variants have been identified, which have circulated globally during the pandemic. Among the seven vaccines produced, the results obtained from the subunit vaccine indicate reliable neutralization levels and protective efficacy. In addition, the high neutralizing antibody levels of subunit vaccines are highly predictive of protective efficacy during SARS-CoV-2 infection [3,4].

The production of recombinant proteins is essential for subunit protein-based vaccines. In the design of many recombinant proteins, specific residues, such as multiple histidine (His) residues, are added to the N- or C-termini of target sequences to facilitate purification and detection. The addition of residues in such small-sized tags is not commonly considered to have an influence on the structure and function of the altered proteins [5]. Nevertheless, there is a rising number of reports showing that this approach may have serious drawbacks [6,7]. Recent evidence indicates that the removal of the His-tag has beneficial effects on the stability of selected proteins [6].

Previous studies have shown that the binding of the spike receptor-binding domain (RBD) protein to the human angiotensin-converting enzyme 2 (hACE2) receptor of host cells is the main pathway of infection [8,9,10]. In this study, we investigated the differences in immunogenicity between the tag-free RBD and His-tag RBD proteins via mouse models. We found that the addition of a polyhistidine tag on the SARS-CoV-2 RBD, when used as a vaccine antigen, may impact protein immunogenicity. Moreover, the differential patterns induced by non-tagged RBD present more rapid and desirable antibody responses when compared with those of His-tag SARS-CoV-2 RBD. Our findings provide important information for antigen design in the development of COVID-19 subunit vaccines.

## 2. Materials and Methods

### 2.1. RBD Protein Production and Purification

Two recombinant SARS-CoV-2 RBD expression plasmids (tag-free or His-tag) covering 333–516 aa of the spike protein were constructed in pSecTag2A. After transfecting the respective plasmids into Expi-293 cells (ThermoFisher, Waltham, MA, USA), the cells were incubated at 37 °C for 5 days, and the secreted RBD proteins were harvested from the culture supernatant. The culture supernatant was first clarified by low-speed centrifugation (2500× *g*, 10 min) and was concentrated using a crossflow device (Sartorius Stedim Biotech, Göttingen, Germany). For the purification of tag-free RBD proteins, a two-step chromatography system comprising a hydrophobic interaction phenyl column (GE Healthcare, Chicago, IL, USA) and a size exclusion column (GE Healthcare) was used. Briefly, after the column was pre-equilibrated with 10 mM Tris–HCl (pH 7.0), the concentrated supernatant was loaded onto the GE AKTA Purifier FPLC system at a flow rate of 1 mL/min. The binding buffer was 1.3 M ammonium sulfate in 10 mM Tris–HCl (pH 7.0), and the elution buffer was 10 mM Tris–HCl (pH 7.0). The obtained tag-free RBD protein was concentrated by using the 3 kDa Amicon filter (Millipore, Burlington, MA, USA). Purification of the His-tag RBD was conducted based on Ni-NTA affinity chromatography, as previously described [11]. Protein samples were quantified using a Pierce BCA Protein Assay Kit (Thermo, Waltham, MA, USA).

### 2.2. SDS-PAGE and Western Blot

For the validation of SARS-CoV-2 RBD expression, 3 µg of protein sample was mixed with a non-reducing buffer (125 mM Tris–HCl, 4% SDS, 0.01% bromophenol blue, and 20% glycerol (pH 6.8)) and was loaded onto gels (TGX Stain-Free™, FastCast™, Acrylamide Kit, 12%, #1610185, Bio-Rad, Hercules, CA, USA) following the manufacturer’s protocols. The proteins were analyzed via electrophoresis for 50–60 min at 145 V in an electrophoresis system (Bio-Rad). For Western blotting, the proteins were transferred onto a 0.22 µm polyvinylidene difluoride PVDF membrane (Bio-Rad, Hercules, CA, USA) using a Trans-Blot transfer system (Bio-Rad). The membrane was blocked with 5% silk milk (Difco, Detroit, MI, USA) and then incubated with a SARS-CoV-2 anti-spike monoclonal antibody (mAb) (1:1000, Sino Biological, 40591-MM42) at 4 °C overnight. The unbound antibody was removed with TBST washes, and the membrane was incubated with the goat anti-mouse IgG HRP conjugate (1:4000, Jackson ImmunoResearch) for 1 h at room temperature. The membranes were washed again and then developed using the Clarity Western ECL Substrate (Bio-Rad, Hercules, CA, USA). Images were captured using the ChemiDoc XRS + System (Bio-Rad).

For antigenicity testing of the immunized mouse serum, recombinant His-tag hemagglutinin (HA) of the avian influenza virus H5N6 (Sino Biological, 40466-V08B, Beijing, China) was used as a His-tag protein that is irrelevant to SARS-CoV-2. In this experiment, 1 µg of tag-free RBD and 1 µg of HA protein were used as the examined antigens. A tag-free RBD or His-tag-RBD-immunized mouse serum (both from day 147 post-immunization) or an anti-His monoclonal antibody (1:1000, Roche, Basel, Switzerland) was used as the first antibody and was incubated overnight at 4 °C. The goat anti-mouse IgG HRP conjugate (1:4000, Jackson ImmunoResearch, West Grove, PA, USA) was used as the secondary antibody and was incubated for 1 h at room temperature. After washes, the membranes were developed using the Clarity Western ECL Substrate (Bio-Rad). Images were captured using the ChemiDoc XRS + System (Bio-Rad).

### 2.3. hACE2 Binding Assay

The binding affinity between hACE2 and tag-free or His-tag RBD proteins was evaluated. Briefly, 200 ng of recombinant hACE2 [11] was coated in a 96-well plate and incubated overnight at 4 °C. Serially diluted recombinant RBD proteins were added to the plate and were incubated for 2 h at room temperature. After washes, an anti-RBD mouse serum (1:4000) was added as the primary antibody and was incubated overnight at 4 °C. Subsequently, a goat anti-mouse IgG HRP conjugate (Jackson ImmunoResearch) was used as the secondary antibody and was incubated for 1 h at room temperature. After further washes, a TMB substrate (KPL) was applied to the plate (100 μL/well) and was incubated in the dark for 10 min, followed by the addition of 1 M H_2_SO_4_ to stop the reaction. All the antibody incubation steps were conducted at room temperature, and a microplate reader (Synergy H1, BioTek, Winooski, VT, USA) was used to read the OD at 450 nm. The 50% effective concentration (EC_50_) was calculated with the OD value, which was determined with seven concentrations (80, 20, 5, 1.25, 0.31, 0.078, and 0.02 μM) to reflect the RBD protein-binding ability.

### 2.4. Animal Study

Five-week-old C57BL/6 female mice were obtained from BioLASCO, Taipei, Taiwan and divided into 4 groups. The mice were subcutaneously immunized with 5 or 15 µg of tag-free or His-tag RBD protein adjuvanted with alum (Alhydrogel, InvivoGen, San Diego, CA, USA) on day 0, 21, and 49. Blood samples were collected on day 0, 21, 35, and 147 from each group. All the animal experiments in this study were reviewed and approved by the Institutional Animal Care and Use Committee (IACUC, approval number: NTU-109-EL-00067).

### 2.5. Antibody Titer

First, 100 ng per well of tag-free RBD was coated onto a 96-well flat-bottom plate (Nunc, Denmark) with the coating buffer (15 mM Na_2_CO_3_ and 35 mM NaHCO_3_ (pH 9.6)) overnight at room temperature. The wells were washed three times with PBST (0.1% (*v*/*v*) Tween 80 in PBS) and blocked with 5% skim milk (Difco) for 1 h. A serially diluted mouse serum was added into the plate (100 μL/well) and was incubated for 1 h. After PBST washes, the goat anti-mouse IgG HRP conjugate (Jackson ImmunoResearch) at 1:2000 dilution was added into the plate (100 μL/well) and was incubated for another hour. After further PBST washes, the TMB substrate (KPL) was applied to the plate (100 μL/well) and was incubated in the dark for 10 min until 1 M H_2_SO_4_ was added to stop the reaction. All the antibody incubation steps were conducted at room temperature. A microplate reader (Synergy H1, BioTek) was used to read the OD at 450 nm.

### 2.6. Neutralization Assay

Neutralizing titers were determined using the live SARS-CoV-2 or pseudotyped SARS-CoV-2. The mouse serum was heat-inactivated (56 °C, 30 min) prior to the neutralization assay. For the live SARS-CoV-2 neutralizing assay, the mouse serum was incubated with 150 PFU of the SARS-CoV-2 Delta variant (B.1.617.2) in the presence of 8 μg/mL TPCK–trypsin of DMEM for one hour at 37 °C. An antibody–virus mixture (200 μL/well) was subsequently added to the confluent Vero E6 cell monolayers, which were pre-treated with 2 μg/mL TPCK–trypsin in the 24-well plates. An hour later, the cells were washed with PBS and then overlaid with 2% (*w*/*v*) methylcellulose in DMEM supplemented with 2% FBS, and then incubated at 37 °C in a 5% CO_2_ incubator. After five to seven days, the cells were fixed with 10% formaldehyde for one hour and stained with 0.5% crystal violet. After washes, the plaques were counted and the data from 50% of the plaque reduction neutralization test (PRNT50) were determined by linear regression analysis. For pseudotyped virus neutralization, a lentivirus that carried the full SARS-CoV-2 spike gene and a defective HIV-1 genome encoding the reporter luciferase was used [11]. A serially diluted mouse serum was incubated with an equal volume of pseudovirus (1000 unit/50 µL) at a final volume of 100 µL at 37 °C for one hour. The mixture was then added to hACE2-expressed 293 T cells in a 96-well plate, and the cells were further incubated for 48 h. The cells were washed once with PBS and were lysed with 50 μL of a cell lysis buffer for 5 min. Following the addition of 50 μL of the luciferase substrate (Luciferase Assay System, Promega) and incubation for 5 min, the luciferase activity was measured using a luminescence microplate reader (Synergy H1, BioTek) and converted into neutralizing titers.

### 2.7. Surface Plasmon Resonance (SPR) for Antibody- and Antigen-Binding Kinetic Assay

Tag-free RBD proteins were immobilized to the CM5 chip (Cytiva, BR100530) using an amine coupling kit (Cytiva, BR100050). Mouse sera immunized with tag-free RBD or His-tag RBD (day 147) were separately used as running modes. The K_D_ values were evaluated through Biacore T200.

### 2.8. Statistics

The data were analyzed with unpaired t tests or an ANOVA, followed by Dunnett’s multiple comparison tests, using Prism 9 (GraphPad Software version 9.4.1, San Diego, CA, USA). Serum titers were calculated based on non-linear regression. *p* values < 0.05 were considered statistically different.

## 3. Results

### 3.1. RBD Protein Expression

**Figure 1:**
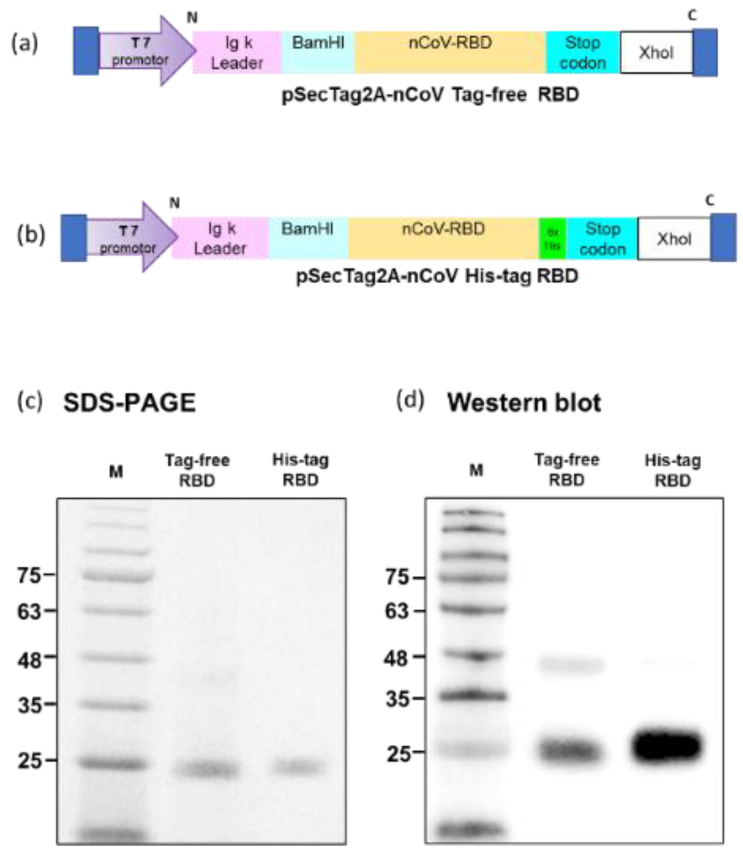
Tag-free RBD and His-tag RBD protein expression. Overview of the tag-free SARS-CoV-2 RBD (**a**) and His-tag SARS-CoV-2 RBD (**b**) construct design in pSecTag2A. The two recombinant SARS-CoV-2 RBD proteins were expressed in Expi293 cells and purified by FPLC. Proteins were analyzed with SDS-PAGE (**c**) and Western blotting with mouse anti-spike monoclonal antibody used for detection (**d**).

Tag-free (Figure 1a) or His-tag (Figure 1b) recombinant SARS-CoV-2 RBD plasmids were constructed in pSecTag2A. The two types of recombinant SARS-CoV-2 spike RBD proteins were produced using the Expi293 mammalian expression system. As indicated in Figure 1c, recombinant SARS-CoV-2 spike RBD proteins were successfully purified and showed the molecular weight of 25 kDa in SDS-PAGE. By using an anti-spike mAb, the signals were clearly detected in the Western blot (Figure 1d). The densitometry intensity ratio of each band for the Western blot is indicated in Figure S1.

### 3.2. hACE2 Binding Affinity

**Figure 2:**
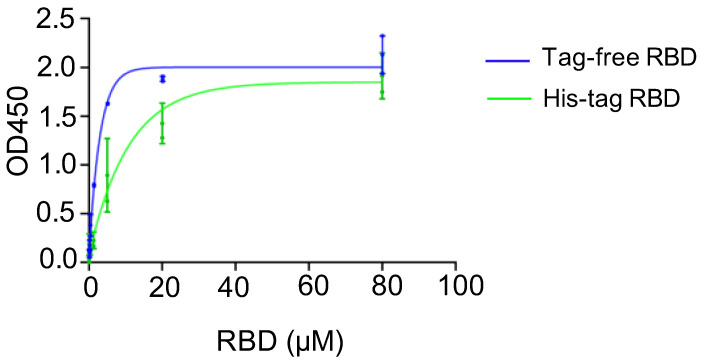
hACE2 binding assay for tag-free RBD (blue) and His-tag RBD (green). Data are presented as mean ± SD.

A binding assay was conducted to investigate the affinity of recombinant SARS-CoV-2 RBD proteins in binding to the hACE2 receptor, and the corresponding kinetics were established. For the two SARS-CoV-2 RBD proteins, the saturated binding concentration was close to 80 μM, and the corresponding OD value was approximately 2.0. Nevertheless, the binding kinetics differed between the tag-free RBD (Figure 2, blue) and His-tag-RBD proteins (Figure 2, green). When the RBD proteins were diluted to 20 μM, the OD450 of the tag-free RBD was 1.9, which was higher than 1.4 for the His-tag RBD. The 50% effective concentration (EC_50_) was 7.51 μM for the His-tag RBD proteins and 1.78 μM for the tag-free RBD proteins, indicating that the tag-free RBD proteins were more effective than the His-tag RBD proteins in binding with hACE2 receptors.

### 3.3. SARS-CoV-2 IgG Antibody Titer and Kinetics

**Figure 3:**
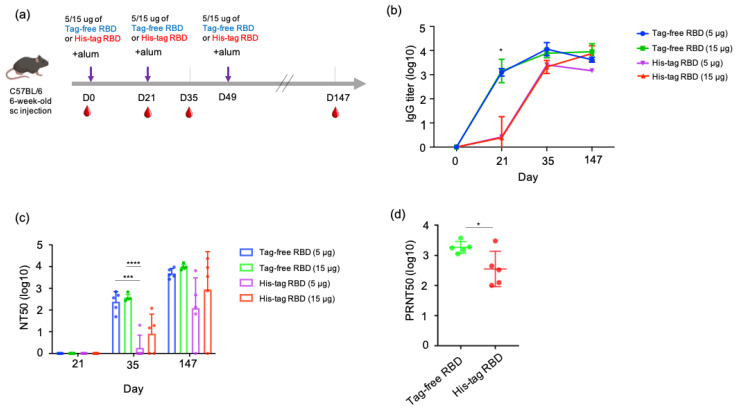
SARS-CoV-2 IgG antibody titers and neutralizing antibodies derived from vaccination with tag-free RBD and His-tag RBD. Design of mouse immunization study (**a**). Antigen-specific IgG titers of mouse serum collected at day 0, 21, 35, or 147 were detected by ELISA (**b**). Neutralization titers of mouse serum collected at day 21, 35, or 147 were detected by pseudotyped virus NT50 assay (**c**). Neutralizing activity of mouse serum collected at day 147 against the Delta variant was determined by live SARS-CoV-2 neutralizing assay (**d**). Data are presented as mean ± SD and results were compared by the Student’s test or ANOVA followed by Dunnett’s test. *p* < 0.5 was considered statistically different. * *p* < 0.05, *** *p* < 0.001, **** *p* < 0.0001.

To understand the antibody kinetics induced by immunization with SARS-CoV-2 proteins, a mouse study was designed, and an illustrated overview is shown in Figure 3a. Sera samples were collected at three time points in the early stage of the experiment (day 0, 21, 35) from groups of mice immunized with either tag-free RBD and His-tag RBD at 15 or 5 µg. Based on the ELISA results, the tag-free RBD mouse groups demonstrated rapid kinetics and a significantly higher titer (1:1418) on day 21, whereas the His-tag-RBD-immunized group showed slower kinetics and a lower titer (1:2.4) (Figure 3b). By day 35 after immunization, the IgG titers from 15 or 5 µg of the tag-free RBD immunization reached 1:7747 or 1:11448, whereas those from 15 or 5 µg of the His-tag RBD immunization had similar titers of approximately 1:1995. At the final observation point on day 147 after immunization, both of the 15 µg groups maintained high titers by 1:8963 (tag-free RBD) and 1:7487 (His-tag RBD), whereas the 5 µg immunization groups showed slightly declined titers, 1:4177 for the tag-free RBD and 1:1454 for the His-tag RBD group.

### 3.4. Comparison of Neutralizing Antibody Responses between Tag-Free RBD and His-Tag RBD Immunization

The neutralizing responses of the mouse serum were determined based on a 50% neutralization titer (NT50) assay. For 21 days after immunization, there was no neutralizing titer observed from samples of all the groups. At 35 days after immunization, 5 µg tag-free RBD induced an average neutralizing titer of 1:240, and 15 µg tag-free RBD induced an average titer of 1:369. However, the His-tag RBD groups of mice developed very low neutralizing titers of 1:1.8 to 1:7.9 at the same time point. In particular, four out of five mice in the 5 ug group and two out of five mice in the 15 ug group did not develop any neutralizing antibodies. Overall, after receiving the second vaccination dose, the tag-free-RBD-immunized mice developed robust virus-neutralizing activity in the serum in the early immunization stage and maintained a high level of neutralizing activity.

After the third dose of vaccination on day 49, we further investigated the longevity of the neutralizing antibody by examining the mouse serum 21 weeks after primary immunization. The mean neutralization titers were 1:4844 and 1:9537 in the 5 and 15 µg tag-free RBD groups, respectively, whereas those of the His-tag RBD groups were only 1:123 and 1:895. More importantly, the His-tag-RBD-immunized groups of mice revealed variable titers, ranging from 0 to 1:6546, and one mouse from the 5 or 15 µg His-tag RBD protein group was found to have no neutralizing antibodies (Figure 3c). Collectively, at day 147 after primary immunization, the tag-free RBD groups were found to have sustained and consistent neutralizing titers in mice.

We further tested the neutralizing activity of the mouse serum derived from 15 ug of tag-free or His-tag RBD immunization against the Delta variant. The results showed that the tag-free-RBD-immunized mouse serum demonstrated greater cross-neutralization of the Delta variant and had a mean titer of 1:1845 (ranging from 1117 to 3741) on day 147, while the His-tag RBD group showed a mean titer of 1:355 with a higher spread in values, which ranged from 101 to 3013 (Figure 3d). The difference between the two groups was significant (*p* < 0.05).

### 3.5. The Molecular Kinetics and Binding Affinity of Tag-Free RBD and Antisera

**Figure 4:**
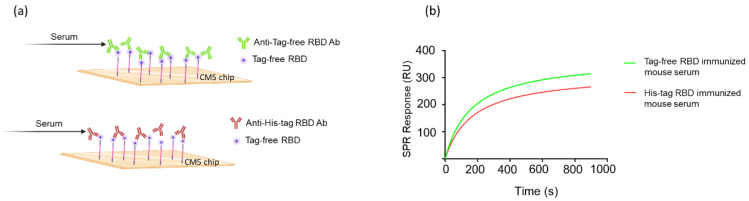
Molecular kinetics and affinity of binding between RBD proteins and mouse antisera. The CM5 chip was immobilized with tag-free RBD proteins, and the mouse antisera were derived from tag-free RBD antigens or His-tag RBD antigens (**a**). The SPR binding curve was modeled using Prism 9.0 (**b**).

To investigate the molecular interaction between tag-free RBD proteins and their induced antibodies, the K_D_ was calculated using Biacore T200 evaluation software to measure the surface plasmon resonance biosensor signal. Additionally, a simple 1:1 interaction with pseudo-first-order kinetics was used to calculate the binding ability (Figure 4a). The binding affinity K_D_ of the tag-free-RBD-immunized mouse sera was 8.2 × 10^−12^ M, whereas His-tag-RBD-immunized mouse sera bound the tag-free RBD proteins with a K_D_ of 1.4 × 10^−10^ M. Therefore, the binding affinity K_D_ of the tag-free-RBD-immunized mouse serum was greater than that of the His-tag-RBD-immunized mouse serum (Figure 4b). These results support the notion that the His-tag RBD protein antigens, rather than the tag-free RBD protein antigens, generate antibodies with less specificity.

### 3.6. Analysis of Mouse Sera Binding with Irrelevant His-Tag Influenza Viral Protein

**Figure 5:**
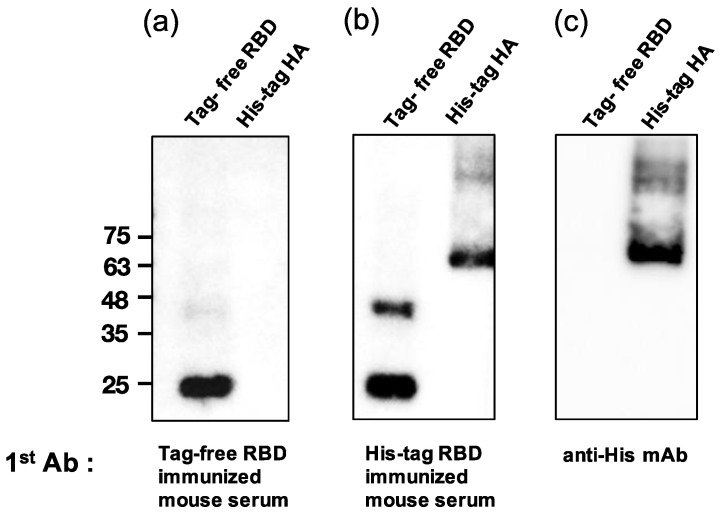
Analysis of mouse sera binding with irrelevant His-tag influenza viral protein. Tag-free RBD and His-tag HA were used as loading antigens and probed with tag-free-RBD-immunized mouse serum (1:800) (**a**), His-tag immunized mouse serum (1:800) (**b**), and the anti-His mAb (1:1000) (**c**).

To examine whether the SARS-CoV-2 RBD-immunized mouse serum contained anti-His antibodies, a Western blot analysis was conducted for both tag-free RBD and irrelevant His-tag HA protein as loading antigens. As shown in Figure 5a, while the tag-free-RBD-immunized mouse serum (day 147, diluted 1:800) recognized its homologous antigen (tag-free RBD, 25 kDa), the serum did not bind the irrelevant His-tag HA derived from the influenza virus, indicating that there were no anti-His antibodies in the serum. However, the His-tag-RBD-immunized mouse serum (day 147, diluted 1:800) not only recognized the tag-free RBD antigen, but also the irrelevant His-tag influenza viral HA antigen (Figure 5b), and the detection of the His-tag on the HA protein by anti-His mAb was evident in the Western blot results (Figure 5c). The densitometry intensity ratio of each band for the Western blot is indicated in Figure S1.

## 4. Discussion

The results of our previous study showed that SARS-CoV-2 RBD induces a neutralizing antibody response [11]. In our study here, we further confirmed that using a tag-free RBD for immunization results in more rapid and potent neutralizing responses to SARS-CoV-2. When the Delta variant was used, the tag-free-RBD-immunized group showed significantly higher titers. The results of recent subunit vaccine studies indicate that reliable neutralization levels are highly predictive of protective efficacy during SARS-CoV-2 infection [3,4]. Hence, robust neutralizing titers are essential for the fight against COVID-19. The antigenic anatomy of the SARS-CoV-2 RBD protein determines the path for protective and therapeutic antibodies [12,13,14]. According to Dejnirattisai et al., the major contribution to virus neutralization was attributed to anti-RBD antibodies, with 55–87% of neutralization due to RBD binders [13]. As many scientists have observed, amino acid changes in the RBD have been implicated in increased viral fitness and potential for immune evasion. Although the RBD accounts for only 2% of the amino acid changes observed in the entire spike protein, it is responsible for more than 90% of the neutralization antibodies generated by humoral responses [15].

The advantages of subunit vaccines include easy manufacturing and transportation and the fact that there is no need for cold chain storage, which is required for mRNA vaccines. Moreover, there is strong evidence from clinical trials demonstrating that RBD-based vaccines are safe and effective against COVID-19 [16]. The addition of a polyhistidine tag to the N- or C-terminus in the protein of interest is very common in recombinant protein technology, and this includes the RBD of SARS-CoV-2. However, there are also reports that the type and position of His-tags influence the protein expression [17]. Additional evidence suggests that His-tag proteins can cause alterations in protein structure, interfering with binding interactions and their picosecond dynamics [18]. More recently, Sandhu et al. [19] and Liu et al. [20] reported that a Flag-tag impacts the protein function and structure. Two review articles have also discussed the negative impacts of those affinity tags, including c-Myc, GST, MBP, etc., on the fusion protein [21,22]. However, few studies have addressed whether a fusion tag influences the immunogenicity of proteins as vaccine antigens. In this study, compared with the His-tag RBD, the tag-free RBD showed a more effective binding affinity with hACE2 receptors based on EC_50_. The SPR system has been used to assess the interaction between SARS-CoV-2 RBD proteins and hACE2 receptors or between SARS-CoV-2 RBD proteins and neutralizing antibodies [23,24]. Therefore, the SPR assay was further used in this study to investigate the affinity of binding between RBD and antibodies. The data suggest that the affinity is higher for binding between RBD proteins and tag-free-RBD-immunized mouse sera than for binding between RBD proteins and His-tag-immunized mouse sera. The tag-free RBD induced rapid and potent IgG titers as early as 21 days after the immunization of mice. The association of SARS-CoV-2 RBD with hACE2 receptors represents the first step required for cellular entry. The higher the affinity of the tag-free RBD in binding hACE2 receptors, the more neutralizing antibodies will be generated. In addition, the greater the number of such antibodies, the more effective their resistance to SARS-CoV-2 infection. The structure between tag-free RBD and His-tag RBD as an antigen is the key element that affects the binding activities and neutralization antibodies. Furthermore, as evidenced by our Western blot results, anti-His antibodies along with anti-RBD antibodies were induced after the immunization of mice. These antibodies directed to the C-terminal His-tag are non-antigen-specific and may impair antigen-specific immune responses. It was reported that an N-terminal but not a C-terminal Flag-tag on TPP1 impaired telomerase function [19]; however, how the position of the His-tag influences the immunogenicity of the RBD awaits further study.

A few months after the discovery of the Delta variant, medical reports showed that it had become the most dominant strain. Subsequently, breakthrough infection cases continued to rise during the later phase of the pandemic. The investigation of viral variants of concern (VOC) has become of interest in public health and biomedical studies worldwide. The recent characterization of the crystal structures of hACE2–RBD complexes helps to identify the key residues facilitating changes in the binding affinity of the hACE2 [25]. This implies that the RBD–hACE2 structure plays a significant role when developing RBD-based subunit vaccines. Another perspective maintains that the binding properties between Omicron RBD and hACE2 have a similar affinity, as does the complex structure of Delta RBD–hACE2 [26]. Our results also indicate that tag-free-RBD-immunized mouse sera harbor potent neutralizing antibodies that can target the Delta variant. Therefore, the tag-free RBD mouse sera may have the same type of effect in neutralizing Omicron variants.

In addition, effective and long-term humoral immunity depends on the processing of germinal centers, where the critical immune cells are located [27]. The follicular dendritic cells, B cells, and T follicular helper cells are all involved in the maturation of promising antibodies [28,29]. When using a His-tag antigen, the immune cells need to process the His-tag and target antigen simultaneously. As a result, the development of neutralizing antibodies might be impaired. This is critical when it comes to the development of subunit vaccines. In our study, the tag-free RBD proteins were purified via a two-step commercial column-based purification, where the protocols were standardized. Through this expression and purification system, recombinant SARS-CoV-2 RBD with an ideal hACE2-binding affinity and desired purity (>95%) may serve as a promising vaccine platform.

## 5. Conclusions

Overall, we demonstrated that the addition of a polyhistidine tag on the SARS-CoV-2 RBD can significantly impair protein immunogenicity, where the tag-free RBD clearly induced a more rapid and robust COVID-19 humoral response than the His-tag RBD in this study. These antibody responses persisted for up to 5 months and demonstrated a greater cross-neutralization of the viral variant. These pivotal points make a strong argument for the preferred use of the tag-free RBD protein as an effective COVID-19 vaccine antigen.

## Data Availability

The data presented in this study are available on request from the corresponding author.

## Supplementary Materials

The following are available online at https://www.mdpi.com/article/10.3390/vaccines10111839/s1, Figure S1: The densitometry intensity ratio of each band for the Western blot.

## References

[1] Jain S., Batra H., Yadav P., Chand S. (2020). COVID-19 Vaccines Currently under Preclinical and Clinical Studies, and Associated Antiviral Immune Response. Vaccines.

[2] Poland G.A., Ovsyannikova I.G., Kennedy R.B. (2020). SARS-CoV-2 immunity: Review and applications to phase 3 vaccine candidates. Lancet.

[3] Keech C., Albert G., Cho I., Robertson A., Reed P., Neal S., Plested J.S., Zhu M., Cloney-Clark S., Zhou H. (2020). Phase 1-2 Trial of a SARS-CoV-2 Recombinant Spike Protein Nanoparticle Vaccine. N. Engl. J. Med..

[4] Khoury D.S., Cromer D., Reynaldi A., Schlub T.E., Wheatley A.K., Juno J.A., Subbarao K., Kent S.J., Triccas J.A., Davenport M.P. (2021). Neutralizing antibody levels are highly predictive of immune protection from symptomatic SARS-CoV-2 infection. Nat. Med..

[5] Hunter M., Yuan P., Vavilala D., Fox M. (2019). Optimization of Protein Expression in Mammalian Cells. Curr. Protoc. Protein Sci..

[6] Booth W.T., Schlachter C.R., Pote S., Ussin N., Mank N.J., Klapper V., Offermann L.R., Tang C., Hurlburt B.K., Chruszcz M. (2018). Impact of an N-terminal Polyhistidine Tag on Protein Thermal Stability. ACS Omega.

[7] Carson M., Johnson D.H., McDonald H., Brouillette C., Delucas L.J. (2007). His-tag impact on structure. Acta Crystallogr. D Biol. Crystallogr..

[8] Wu Y., Wang F., Shen C., Peng W., Li D., Zhao C., Li Z., Li S., Bi Y., Yang Y. (2020). A noncompeting pair of human neutralizing antibodies block COVID-19 virus binding to its receptor ACE2. Science.

[9] Yang J., Wang W., Chen Z., Lu S., Yang F., Bi Z., Bao L., Mo F., Li X., Huang Y. (2020). A vaccine targeting the RBD of the S protein of SARS-CoV-2 induces protective immunity. Nature.

[10] Yang Y., Du L. (2021). SARS-CoV-2 spike protein: A key target for eliciting persistent neutralizing antibodies. Signal Transduct. Target. Ther..

[11] Huang P.H., Tsai H.H., Liao B.H., Lin Y.L., Jan J.T., Tao M.H., Chou Y.C., Hu C.J., Chen H.W. (2021). Neutralizing antibody response elicited by SARS-CoV-2 receptor-binding domain. Hum. Vaccines Immunother..

[12] Clark S.A., Clark L.E., Pan J., Coscia A., McKay L.G.A., Shankar S., Johnson R.I., Brusic V., Choudhary M.C., Regan J. (2021). SARS-CoV-2 evolution in an immunocompromised host reveals shared neutralization escape mechanisms. Cell.

[13] Dejnirattisai W., Zhou D., Ginn H.M., Duyvesteyn H.M.E., Supasa P., Case J.B., Zhao Y., Walter T.S., Mentzer A.J., Liu C. (2021). The antigenic anatomy of SARS-CoV-2 receptor binding domain. Cell.

[14] Hurlburt N.K., Seydoux E., Wan Y.H., Edara V.V., Stuart A.B., Feng J., Suthar M.S., McGuire A.T., Stamatatos L., Pancera M. (2020). Structural basis for potent neutralization of SARS-CoV-2 and role of antibody affinity maturation. Nat. Commun..

[15] Chen C., Boorla V.S., Banerjee D., Chowdhury R., Cavener V.S., Nissly R.H., Gontu A., Boyle N.R., Vandegrift K., Nair M.S. (2021). Computational prediction of the effect of amino acid changes on the binding affinity between SARS-CoV-2 spike RBD and human ACE2. Proc. Natl. Acad. Sci. USA.

[16] Falke B.A. (2000). Purification of Proteins Using Polyhistidine Affinity Tags. Methods Enzymol..

[17] Paul N.K., Baksh K.A., Arias J.F., Zamble D.B. (2020). The impact of a His-tag on DNA binding by RNA polymerase alpha-C-terminal domain from Helicobacter pylori. Protein Expr. Purif..

[18] Thielges M.C., Chung J.K., Axup J.Y., Fayer M.D. (2011). Influence of histidine tag attachment on picosecond protein dynamics. Biochemistry.

[19] Sandhu R., Wei D., Sharma M., Xu L. (2019). An N-terminal Flag-tag impairs TPP1 regulation of telomerase function. Biochem. Biophys. Res. Commun..

[20] Liu X., Liu L., Bi W., Alcorn J.L. (2020). An internal amino-terminal FLAG-tag octapeptide alters oligomerization of expressed surfactant protein-A. Protein Expr. Purif..

[21] Zhao X.Y., Li G.S., Liang S.F. (2013). Several Affinity Tags Commonly Used in Chromatographic Purification. J. Anal. Methods Chem..

[22] Kimple M.E., Brill A.L., Pasker R.L. (2013). Overview of affinity tags for protein purification. Curr. Protoc. Protein Sci..

[23] Mercurio I., Tragni V., Busto F., De Grassi A., Pierri C.L. (2021). Protein structure analysis of the interactions between SARS-CoV-2 spike protein and the human ACE2 receptor: From conformational changes to novel neutralizing antibodies. Cell Mol. Life Sci..

[24] Raghu D., Hamill P., Banaji A., McLaren A., Hsu Y.T. (2022). Assessment of the binding interactions of SARS-CoV-2 spike glycoprotein variants. J. Pharm. Anal..

[25] Han P., Su C., Zhang Y., Bai C., Zheng A., Qiao C., Wang Q., Niu S., Chen Q., Zhang Y. (2021). Molecular insights into receptor binding of recent emerging SARS-CoV-2 variants. Nat. Commun..

[26] Han P., Li L., Liu S., Wang Q., Zhang D., Xu Z., Han P., Li X., Peng Q., Su C. (2022). Receptor binding and complex structures of human ACE2 to spike RBD from omicron and delta SARS-CoV-2. Cell.

[27] Sokal A., Chappert P., Barba-Spaeth G., Roeser A., Fourati S., Azzaoui I., Vandenberghe A., Fernandez I., Meola A., Bouvier-Alias M. (2021). Maturation and persistence of the anti-SARS-CoV-2 memory B cell response. Cell.

[28] Heesters B.A., Myers R.C., Carroll M.C. (2014). Follicular dendritic cells: Dynamic antigen libraries. Nat. Rev. Immunol..

[29] Van der Poel C.E., Bajic G., Macaulay C.W., van den Broek T., Ellson C.D., Bouma G., Victora G.D., Degn S.E., Carroll M.C. (2019). Follicular Dendritic Cells Modulate Germinal Center B Cell Diversity through FcgammaRIIB. Cell Rep..

