# Supplementary material for: Tag-Free SARS-CoV-2 Receptor Binding Domain (RBD), but Not C-Terminal Tagged SARS-CoV-2 RBD, Induces a Rapid and Potent Neutralizing Antibody Response
Source: Vaccines (Basel). 2022 Oct 30;10(11):1839. doi: 10.3390/vaccines10111839 (PMC9692485; doi:10.3390/vaccines10111839)
Supplement: Supplementary file 1 [file vaccines-10-01839-s001.zip › vaccines-1968055-supplementary.pptx]

## Slide 1
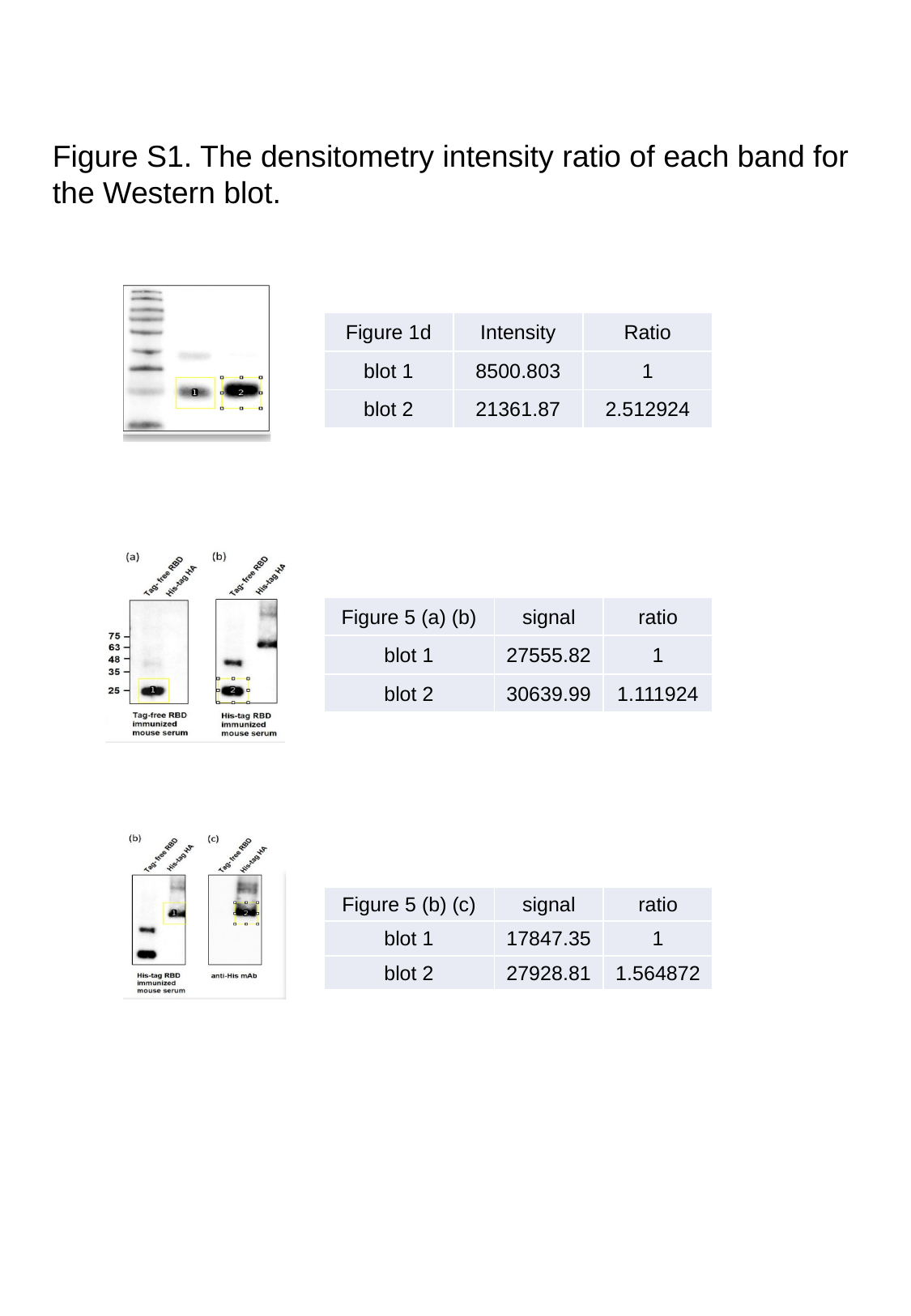

Figure S1. The densitometry intensity ratio of each band for the Western blot.
| Figure 1d | Intensity | Ratio |
| --- | --- | --- |
| blot 1 | 8500.803 | 1 |
| blot 2 | 21361.87 | 2.512924 |
| Figure 5 (a) (b) | signal | ratio |
| --- | --- | --- |
| blot 1 | 27555.82 | 1 |
| blot 2 | 30639.99 | 1.111924 |
| Figure 5 (b) (c) | signal | ratio |
| --- | --- | --- |
| blot 1 | 17847.35 | 1 |
| blot 2 | 27928.81 | 1.564872 |
